# Nature Isn’t What It Used To Be

**DOI:** 10.3201/eid1505.000000

**Published:** 2009-05

**Authors:** Polyxeni Potter

**Affiliations:** Centers for Disease Control and Prevention, Atlanta, Georgia, USA

**Keywords:** Art science connection, emerging infectious diseases, art and medicine, Alexis Rockman, biotechnology, hyperrealism, foodborne diseases, farm animals, about the cover

**Figure:**
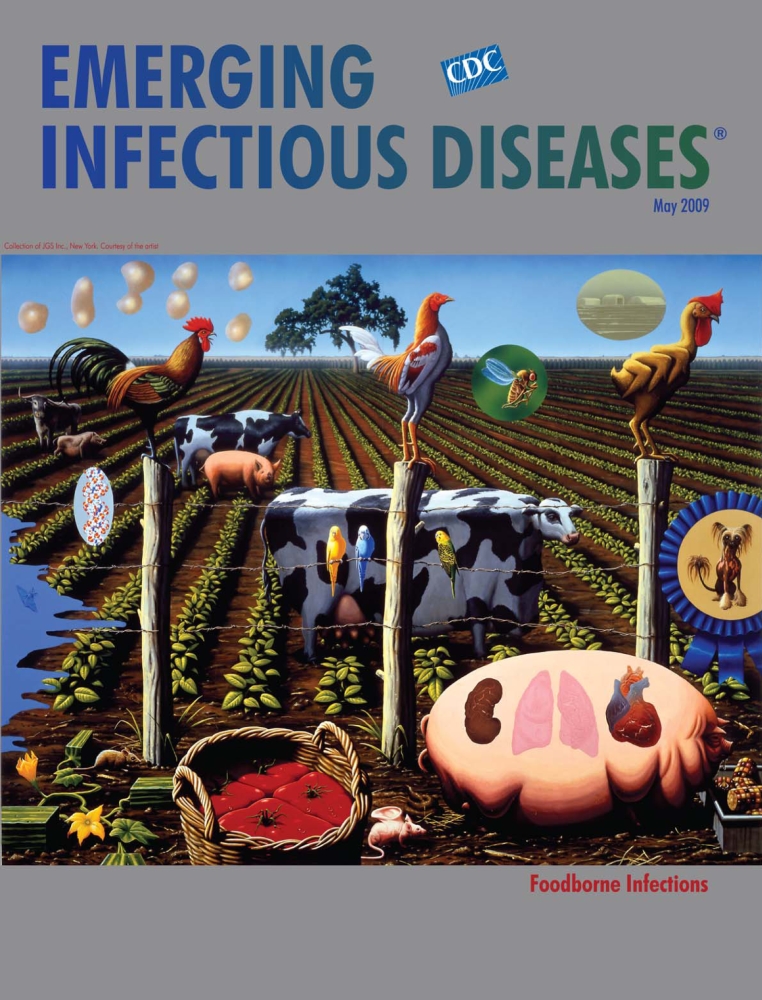
Alexis Rockman (b. 1962) The Farm (2000) Oil and acrylic on wood (243.84 cm × 304.8 cm) Collection of JGS, Inc. Courtesy of the artist

“There are also cocks, which are extraordinary size, and have their crests not red as elsewhere, or at least in our country, but have the flower-like coronals of which the crest is formed variously colored,” wrote traveler and geographer Megasthenes 17 hundred years ago. “Their rump feathers are neither curved nor wreathed but are of great breadth and they trail them in the way peacocks trail their tails, when they neither straighten nor erect them: the feathers of these Indian cocks are in color golden and also dark-blue like the smaragdus.” These impressive cocks, and other fantastic creatures, populated *Ta Indica*, the author’s account of India. Megasthenes’ flamboyant beasts, some of them sporting nonstandard digits and extra heads, may have come from the stories of people he met in his travels and not his own observations. Nonetheless, they could be describing the creatures of Alexis Rockman, artist, naturalist, author, educator, activist.

“I try to use all of the ways we depict nature and natural history as content,” Rockman says, explaining the natural sciences bias of his subjects. “I’m interested in credibility.” His love for science and art flourished during childhood in his native New York. “I grew up in the American Museum of Natural History. My mother was an assistant to Margaret Meade … it shaped my perspective. ... Charles R. Knight and Chesley Bonestell were my heroes.” His education at the School of Visual Arts complemented long studies of nature in the United States and abroad. “I started out thinking that I would be a scientist. Eventually, over the years, I ended up becoming interested in other types of practices, like certain genres of filmmaking, animation. I think what I ended up doing was really a combination of all those different interests. I’ve always been interested in the history of the representation of nature.”

Rockman’s style, now realistic now abstract, has eluded traditional descriptions, “I try to make it as credible as possible without making it boring.” Some have seen surrealism in his unusual depictions of plants, animals, and humans, “What I am after … is the disturbing part or the transformative part.” Embracing popular culture, he flaunts it with great precision in a manner called hyperrealism in the tradition of Grant Wood, who captured the rural Midwest of the 20th century, especially in his American Gothic. Like pop art icon Andy Warhol, he is comfortable with modern technology and skillful with its agility and interactivity, “computer-manipulated, archetypal images that we’ve all seen, or if we haven’t seen them we feel that we know them.” The Farm, on this month’s cover, was commissioned by Creative Time, a public arts organization, as a New York City billboard. “I like to put people off-kilter by breaking up expected visual patterns.”

The constant struggles between nature and its creatures engage all of Rockman’s interests from evolution, climate change, and genetic engineering to the failures of technology. Like his idol H.G. Wells, the artist portrays humans as, “the unnatural animal, the rebel child of nature,” that, “more and more … turns himself against the harsh and fitful hand that reared him.”

The Farm portrays this favorite theme. Developing it follows the artist’s usual path of discovery, which involves learning from an expert, in this case a molecular biologist, about genetics and artificial selection. The subject drives the medium. The story unfolds with the clarity of a high-definition screen. A field of soybeans extends as far as the eye can see; against it, an allegorical tableau, in Rockman’s words, “the way humans have altered their landscape.”

“The way I constructed it is that, as in a lot of Western culture, we read things from left to right,” he explained in an interview. “On the left side of the image are the ancestral species of the chicken, the pig, the cow, and the mouse”; on the right, their contemporary versions. Farther to the right are “permutations of what things might look like in the future.” The transition from wild cow and boar to familiar barnyard beasts to grotesque technologically engineered models is precise and tactical, and the animals still maintain some original species characteristics. A fruit fly, a strand of DNA, an overmanipulated dog inside a prize-winning blue rosette compete for attention with the cocks placed conspicuously on the fence, straddling the horizon, challenging Megasthenes. Tomatoes created to fit the shipping crate, loaf-shaped watermelons, and multicolor corn complete the picture, occupying several layers of time and genetic activity in the permissive context the artist calls “democratic space.”

Rockman’s vision of biotechnology is riddled with clues and inside jokes rooted in economic, social, ethical, and other concerns. It’s a vision he wants to popularize, “It has to be decipherable to a six-year-old child. I try to construct it as an onion with different layers of meaning and iconography.” The Farm succeeds in this regard, perhaps even beyond the artist’s intentions. This icon of biotechnology is also the stage for foodborne disease emergence. The soybean farm is much too close to the farm animals, whose fertile waste deposits seep into the nearby water used to irrigate the plants. The fence is useless for keeping out rodents or birds and their microbial deposits. And human manipulation, intended to make larger more efficient food animals, may have unintended consequences. If the DNA strands were animated, they would be turning wildly.

A 2006 multistate outbreak of *E*. *coli* O157:H7 infection was associated with salad. Samples taken from a stream, cattle manure, and feces from wild pigs on ranches in Salinas Valley, California, implicated spinach, but after this outbreak, leafy greens were seen as subject to this type of contamination. Rockman’s iconic overlap of urban, agricultural, and cattle-raising elements in ecosystems containing sigmodontine rodents, which are reservoirs of hantaviruses, is a recipe for hantavirus pulmonary syndrome. The syndrome, now found throughout the United States, is rare but deadly. Mad cow disease emerged in Great Britain, possibly as an interspecies transfer of scrapie from sheep to cattle and moved around the globe through trade. Spread from human to human is now a threat through contaminated hospital equipment and blood transfusions. People who consume antler velvet as a nutritional supplement may also be at risk for exposure to prions.

“Nature isn’t what it used to be,” Rockman wrote in one of his books. And of course it never was or ever will be. But the irony of that statement awaits future developments. Because, as H.G. Wells put it, “The only true measure of success is the ratio between what we might have done and what we might have been on the one hand, and the thing we have made and the things we have made of ourselves on the other.”
